# Family Functioning and Emotional Aspects of Children with Autism Spectrum Disorder in Southern Brazil

**DOI:** 10.1007/s10803-022-05497-z

**Published:** 2022-03-17

**Authors:** Tatiana M. N. Flenik, Tiago S. Bara, Mara L. Cordeiro

**Affiliations:** 1 Faculdades Pequeno Príncipe, Curitiba, Brazil; 2 Instituto de Pesquisa Pelé Pequeno Príncipe, Av. Silva Jardim, 1632, Curitiba, PR 80250-200 Brazil; 3 Department of Psychiatry and Biobehavioral Sciences, University of California Los Angeles, Los Angeles, CA USA

**Keywords:** Autism spectrum disorder, Family relationships, Stress, Family Environment Scale, Strengths and Difficulties Questionnaire, Questionnaire on Resources and Stress—Short Form

## Abstract

Autism spectrum disorder (ASD) affects children and their families. We investigated the family environment of children with ASD in Brazil. Twenty-one families participated. Outcome measures administered to parents included the Strengths and Difficulties Questionnaire, Questionnaire on Resources and Stress—Short Form, and the Family Environment Scale. All families reported a number of difficulties, including social interactions and peer relationships, stress levels, and communication difficulties. Families also reported great concern for their child’s future. Brazil is a developing country, yet there are few national resources and studies related to ASD. The challenges continue from diagnosis through treatment. Our results emphasize the need to understand the family environment of children with autism and their parents’ apprehensions to develop effective coping programs.

Autism spectrum disorder (ASD) is a neurodevelopmental disorder that affects social, behavioral, and communication development. One out of every 54 school-age children has ASD (APA, 2013; Maenner et al., 2020). In Brazil, only one study explored prevalence of ASD, showing a rate of 1 in 368 school-age children (Paula et al., 2011). In Brazil’s southern region, prevalence of 1 case per 2597 school-age children is estimated (Beck, 2017). The ASD diagnosis is based on clinical observations following Diagnostic and Statistical Manual of Mental Disorders-5 (DSM-5) guidelines (APA, 2013): socio-communicative deficits, presence of repetitive and stereotyped behaviors, isolated interests, and sensory abnormalities (hyposensitivity or hypersensitivity) (APA, 2013). Post diagnosis, effective treatments and interventions can facilitate a better prognosis, developing efficient adaptive behaviors, and learning strategies to cope with challenges. Most children improve when they receive proper care and early intervention with effective results. (Bosa, 2006; Lampreia, 2007). Yet finding such treatments and interventions may be challenging for affected individuals and their families, especially for Brazilian families, due to limited access to specialized health and social support services (Gomes et al., 2015; Listik & Listik, 2020).

Previous studies have determined that certain characteristics of the family system are essential for a child’s healthy development (Sameroff, 1990; Vianna et al., 2007). Therefore, understanding family profiles of children and adolescents diagnosed with ASD can help in providing adequate and efficient treatments. These families are more likely to exhibit symptoms of stress and a reduced quality of life. Through a comprehensive family profile, it is possible to establish effective support and address important unmet needs, which sustain continuity of better quality of life and treatment benefits (Koly et al., 2021; Leonardi et al., 2021; Musetti et al., 2021; Park, 2021).

In 1965, parents of children with ASD formed the world's first association on autism, La National Society for Autistic Children. Consequently, parents' associations have contributed to raising societal awareness, providing more information about autism, and seeking greater support and rights for children with special needs from public agencies (Leandro and Lopes 2018). Despite the work of such dedicated associations in Brazil, few authors have addressed specific aspects related to autism and its impact on the families of children with ASD in Brazil (Leandro and Lopes 2018). Given Brazil’s geographic and cultural extensiveness, multi-faceted studies would be necessary to address cultural diversity. This is especially the true in terms of evaluating how various diagnostic strategies are applied across the country (Miele & Amato, 2016). Furthermore, very few existing studies have examined quality of life and stress levels of caregivers and families of children with ASD in Brazil (Miele & Amato, 2016).

The family environment has a great impact on the development, worsening, or improvement of diseases in its members. Changes in daily family environment as well as the need to adapt to different life situations influence family members, and show the importance of parental and family support (Hall & Graff, 2011; Vianna et al., 2007).

Additionally, early intervention with children identified as having ASD from 18 months of age contributes to the quality of life of these individuals and their caregivers and family members, enabling agile and timely interventions by pediatricians, psychologists, and special education teachers (Beck, 2017).

Parents play a significant role in their children's development. Parenting a child with ASD can be stressful and demanding due to the substantial amount of energy, time, and financial investment required to promote the child’s healthy development. Studies have shown that parents of children with ASD generally have more associated anxiety, depression, and other health problems compared to parents of children with neurotypical development patterns (Baixauli et al., 2019).

Parents of a child with ASD also face with parental demands that are different from those encountered by parents of typically developing children. The severity of the child’s ASD symptoms directly affect these demands. Parental stress and the dysfunctional state of family coordination are directly influenced by the severity of symptoms. It is, therefore, important to identify and understand factors directly affecting the child's developmental capacity, especially when there are difficulties accessing specialized therapies due to high costs or a lack of availability of specialized services. It is also important to clarify which are the most taxing demands on the parents residing in countries with fewer available resources (Downes & Cappe, 2021; Ku et al., 2019; Malik-Soni et al., 2021; Mazzoni et al., 2018; McKee et al., 2020; Schall, 2000).

This study investigated the ASD’s impact on the family environment of diagnosed children in developing countries. Additionally, it investigated the emotional environment of children with ASD and their parents. In doing so, we hope to contribute to the scarce literature in developing countries, increase the existing knowledge base and, ultimately, facilitate and promote better coping and intervention programs for families of children with autism.

## Methods

### Participants

We conducted the survey during the SARS COVID-2 pandemic. At the time, the Clinic Self Center was one of the only clinic that agreed to participate in the study. Initially, 90 children received services at the clinic, and through the inclusion criteria, the final sample size was 30 participants, with an estimated sampling error of 19%. Of families invited to participate in the study, 21 with children previously diagnosed with ASD agreed to participate. The participating families were receiving ongoing treatment from a private clinic that specializes in ASD intervention, in a city in southern Brazil.

The inclusion criteria were as follows: (a) participants should be parents and/or legal guardians of children diagnosed with ASD, (b) children have been formally diagnosed with ASD for at least 3 years, (c) parents/legal guardians are literate, and (d) families should be residents of the city of [blinded for review]. All participating families agreed to complete the required questionnaires for analysis. Informed Consent Form (TCLE) was obtained from them. The main respondent was the child’s mother (95%). The exclusion criteria were as follows: (a) children with a recent ASD diagnosis (< 2 years), (b) parents in psychiatric treatment, and (c) parents unable to read and write.

The research was approved by the Ethics Committee for Research on Human Beings of Faculdades Pequeno Principe, Curitiba, Brazil.

### Measures

Families (n = 21) of children with ASD responded to three questionnaires at the therapeutic clinic where they received daily treatment from a multidisciplinary team.

The questionnaires were the Family Environment Scale (FES) developed by Moos and Moos (1994), the Strength and Difficulties Questionnaire (SDQ) developed by Goodman (1999), and the Questionnaire on Resources and Stress – Short Form (QRS-F), developed by Jean Holroyd in 1974 (Russell et al., 2013; Vianna et al., 2007).

The SDQ is a dimensional measure of psychopathology among children aged 4 to 16 years. It is widely used as an internationally standardized instrument to measure and assess aspects of behavior and externalizing and internalizing symptoms (Goodman, 1999). It is used in research and clinical practice and has been translated in and validated in over 40 languages (Goodman, 1999; Russell et al., 2013). The SDQ consists of 25 items, distributed across five subscales of five items each: prosocial behavior (a measure of the child's ability to be considerate, share, be helpful, and be kind to younger children), hyperactivity/inattention, emotional symptoms, conduct problems, and peer relationship problems subscales (Cury & Golfeto, 2003; Russell et al., 2013).

The FES is a 90-item inventory with 10 subscales. It gathers information on family functioning across domains that access interpersonal relationships, personal growth, and system maintenance (Moos & Moos, 1994). It is also useful for assessing the impact of counseling and other interventions (Moos & Moos, 1994; Vianna et al., 2007). Its 10 subscales are divided into three categories: personal interrelationship (cohesion, expressiveness, and conflict), personal growth (independence, achievement, intellectual/cultural, moral/religiosity), and system maintenance (organization and control). The internal consistency (Cronbach's alpha) ranged between 0.61 and 0.78 for all subscales (Moos & Moos, 1994).

The QRS-F has been used previously to assess possible stressors experienced by family caregivers of children with disabilities, chronic diseases, or developmental disorders (Zanfelici et al., 2016).

### Statistical Analysis

Sample characteristics were summarized using descriptive statistics and measures of central tendency. The data referring to the scales met the cutoff scores established by each scale. Data were analyzed using SPSS software for Windows version 21.0 (IBM Corp., 2012) Statistical significance was set at p < 0.05.

## Results

### Socio-demographic Profile

Table 1 summarizes demographic characteristics of the participants. The ages of the children (14 males and 7 females) were 7.48 ± 3.98 years. The age at diagnosis of ASD varied between 1 year and 6 months to 7 years, with clinical presentations ranging from mild to moderate; there were no presentations of severe ASD in the sample.

**Table 1:** Socio-demographic characteristics of the participants

Variables	n	Average	SD	%
Sex. Male	14	–	–	66.67
Age		7.48	3.98	–
Male	14	7.86	4.31	–
Female	7	6.71	3.40	–
Diagnostic age		5.59	6.54	–
Male	14	5.00	5.87	–
Female	7	6.68	8.02	–
Parental age	21	43.24	5.63	–
Mother		42.10	4.38	–
Father		44.5	6.67	–
Parental education	21	7.34	1.44	–
Mother		7.75	1.21	–
Father		6.89	1.57	–
Respondent	21	–	–	–
Mother	20	–	–	95.24
Father	1	–	–	4.76
Siblings without ASD	9	–	–	–
ASD in family^a^	4	–	–	–

*SD* standard deviation

^a^Cousins who were not living with the study families

The age of mothers ranged between 42.10 ± 4.38 years, and that of fathers ranged between 44.5 ± 6.67 years. Most parents were married (81%). Nine siblings without autism lived with participating families. In terms of extended family composition, it was reported that there were four cousins (from second to fourth degree of kinship) who did not reside with participant families.

### Emotional and Behavioral Characteristics of Children

**Fig. 1:**
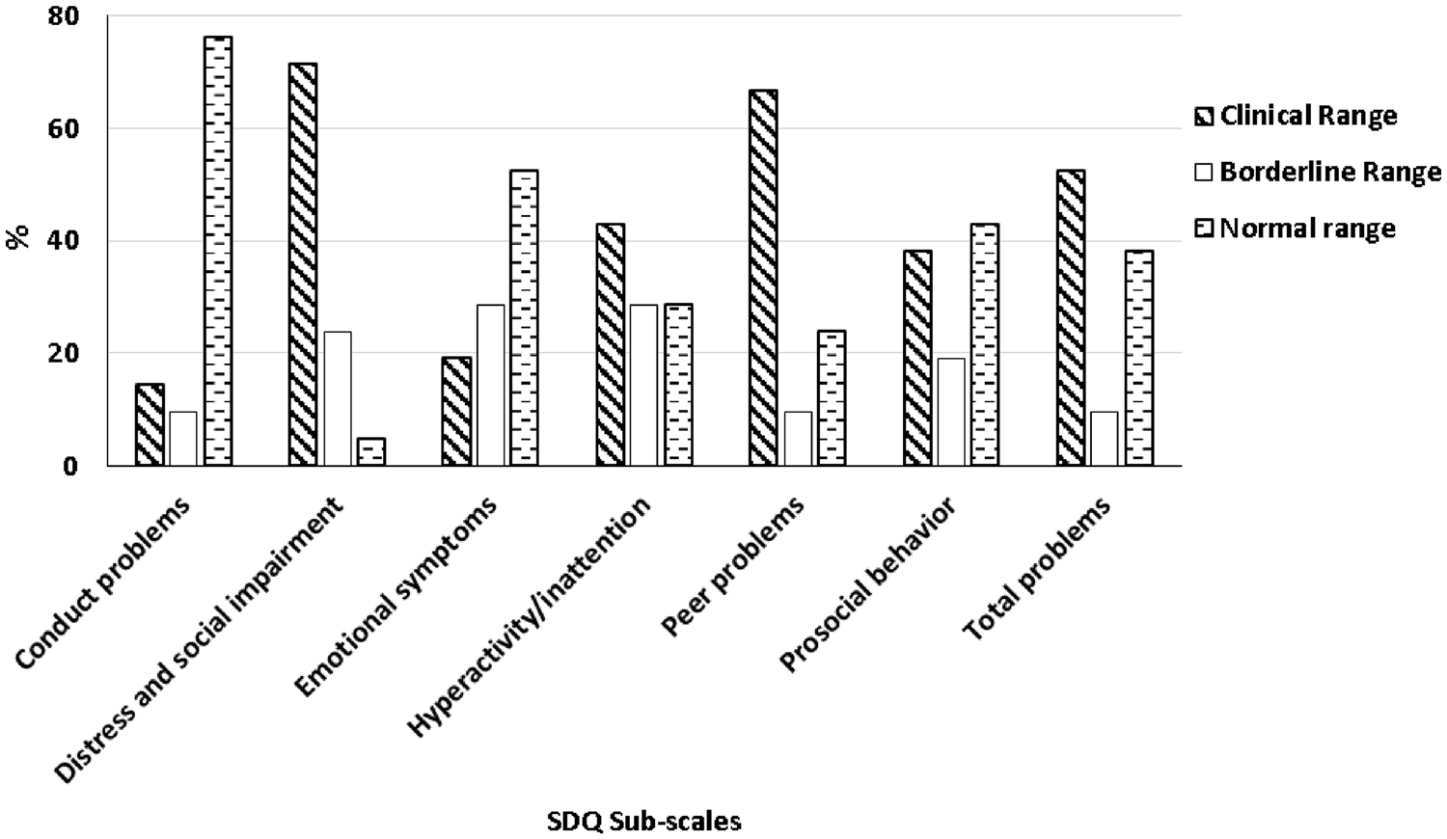
Emotional and behavioral characteristics of the children with ASD according to the Strenght Difficulties Questionnaire (SDQ)

Figure 1 presents the SDQ results of the participants. In the sample, 52.4% experienced problems with peer relationships, 67% displayed stress and social impairment, 14% had conduct problems, and 19% had emotional symptoms.

### Characteristics of the Family Environment

**Fig. 2:**
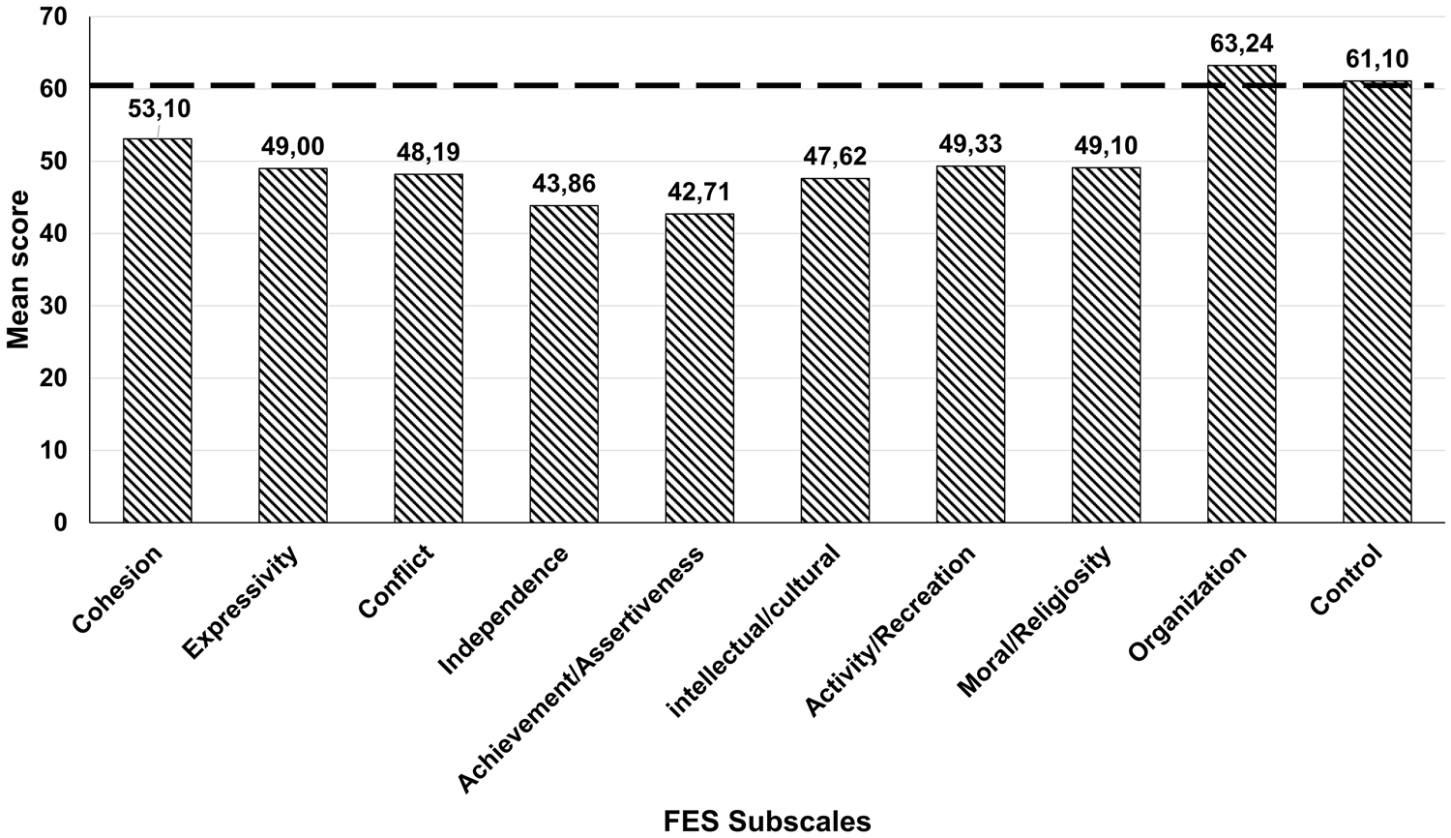
Mean subscale scores of the family environment scale for ASD families

Figure 2 summarizes characteristics related to the sample's family environment, evaluated using the FES. The families presented the following characteristics: low cohesion, low expressiveness, low conflict, and low independence. They were active in intellectual and religious activities, but they tended to have difficulties with organization and control.

### Stress Assessment of Family Caregivers

Of the 31 items that the QRS addresses, we obtained results from 11, which are highlighted in Table 2.

**Table 2:** List of QRS-F statements and percentage of stress-related characteristics

Item	Question	%	
		True	False
4	I worry about what will happen to him/her when I can no longer take of him/her	90.48	9.52
6	He/she has limitations on the type of work he/she can do to support himself	57.14	42.86
7	I accepted the fact that he/she might have to live his/her life somewhere special	23.81	76.19
9	I gave up on things I really wanted to do to take care of him/her	71.43	28.57
23	It is difficult to talk to him/her because he/she has difficulty understanding what is being said to him/her	52.38	47.62
31	I find it easy to relax	33.33	66.67
32	I worry about what will happen to him/her when he/she gets older	90.48	9.52
43	I often worry about what will happen to him/her when I can no longer take care of him/her	71.43	28.57
44	People cannot understand what he/she is trying to say	57.14	42.86
45	Taking care of him/her requires constant effort	76.19	23.81
51	I get worried most of the time	66.67	28.57

Most parents (71%) reported having renounced their plans and personal dreams to care for and encourage their children with ASD.

For working mothers who have children with disabilities, less time was being spent on leisure activities and more was devoted to socialization activities. Despite this, as shown by the QRS, socialization was impaired, as many families reported avoiding engaging activities outside the home, taking walks, and having an active social life (57%).

Communication difficulty was a recurrent theme in the responses of families, as was the diminished social, affective, and peer interaction. This difficulty appeared in 52% of the participant responses, especially in relation to children not understanding their parents. Additionally, around 67% respondents reported difficulty finding time to relax.

## Discussion

This study investigated characteristics related to family environment, stress, and emotional aspects of parents and their children diagnosed with ASD in a developing country. Having a child diagnosed with ASD may impact several dimensions of family functioning and change the family routine. Family relationships are vital for the mental health of children (Phua et al., 2020). Parents also experience various levels of stress due to their child’s diagnosis (e.g. Gomes et al., 2015; Nik Adib et al., 2019). Our study is not representative of Brazil in its entirety, but of a portion of the southern region, where most inhabitants are of European descent, yet it may provide insight that is useful for assisting affected families among the entire population of the country (IBGE, 2010). Despite the importance of exploring the functioning of these families, there have been no previous studies in Brazil that investigated the family environment of children with ASD. In our study, the main family characteristics observed with the FES were low cohesion, expressiveness, assertiveness, and conflict and high organization, with several aspects associated with the level of general stress and social impairment in children (71%). Although literature is scarce regarding analyzing the family environment of children with ASD, our study findings align with those of previous research in which families of children with ASD had low cohesion and ongoing conflict (Kelly et al., 2008). Further, studies that used the FES to assess families of children with ASD according to the DSM Fourth Edition-Text Revision (APA, 2000), demonstrated that families had low expression of feelings and high levels of organization, in addition to low social interaction (Heiman & Berger, 2008).

In this study, parents reported high levels of behavioral and emotional distress in their children with ASD, as seen through the SDQ. Children presented high rates of general stress, and peer interaction problems (71.43%). A previous study has also reported children's behavioral problems and the relationship between symptom severity and peer relationship problems (Haney et al., 2018). Additionally, parents of children with ASD had very high levels of stress, likely due to the challenging behaviors that children presented. Notably, the children also showed elevated stress levels.

The stress levels found in this study are similar to those in other studies reporting elevated stress in mothers of children with ASD (Sprovieri & Assumpção Jr, 2001). Families and guardians of children with ASD also showed a greater level of concern for their well-being when they were no longer able to provide for their child’s needs (Miele & Amato, 2016). In the present study’s sample, a large proportion of families (90%) expressed concern about their children's future, in relation to how the children would support themselves in case of death of the parents.

It was also observed that most families have concerns about: “where their children will live” and “how they will live” with 76% not being able to accept that their children would have to live in a special facility. Almost 90% of the family members were concerned about the future of these children as they themselves aged. Additionally, 71% of mothers and fathers gave up their own plans and dreams to care for their children with ASD. A study of working mothers who had children with a disability indicated that less time was spent on leisure activities while more was spent on socialization activities, perhaps reflecting the need for families to connect to social life and seek support (Smith et al., 2010). These mothers also reported less time for self-care compared to those of children without disabilities (Smith et al., 2010).

Parental stress can be further intensified by communication difficulties. Parents may fear that they do not correctly decode the needs expressed by their child or that they are not well understood by them (Sénéchal & Des Rivières-Pigeon, 2009). A study showed that mothers had a poor quality of life if they had problems related to communication, behavior, and interests of their children with ASD (Øien & Eisemann, 2016). Communication was impaired in half of the participants. Parents reported that it was challenging to talk to the child due to communication difficulties, which is one of the cardinal symptoms of ASD (APA, 2013).

As mentioned above, a high level of stress was perceived in this study’s participants. The difficulty of families with few moments of leisure, socialization, and relaxation became apparent. Results showed that 67% of participants had difficulty relaxing. In another study with 166 parents of children diagnosed with autism, a correlation was found between the severity of autism symptoms and levels of parental stress and depression (Haney et al., 2018).

The dynamics in families of children with ASD can negatively affect the mental and emotional health of the group members. A study found that fathers had high levels of stress while mothers even had higher scores measured with the same scale. Mothers are often responsible for caring for children with ASD. They spend the majority of their time doing so and may therefore experience increased stress and emotional dysfunction (Baixauli et al., 2019; Gray, 1997; Sprovieri & Assumpção Jr, 2001).

Additionally, families of children with ASD showed high levels of stress, and parents of children with ASD showed a higher rate of depression symptoms compared to those of children with other disorders (Kelly et al., 2008). The stress of parents of children with ASD is greater than that of parents of children with neurotypical development (Baixauli et al., 2019). This difference may be related to diagnostic characteristics of children with ASD, such as the severity of disability, child's age, and the extent of the behavioral problems coexisting with comorbid conditions.

The limitations of the study must be addressed. First, it had a small number of participants, which not allow for statistical correlation analyses. Second, a control group of families with typically developing children was not used. Finally, children with severe or moderate ASD were excluded from the study. However, it was possible to assess and obtain a profile characteristic of the family environment of children diagnosed with ASD. Further research should be done with a larger number of participants recruited from all regions of Brazil. A comparison group of typically developing children should also be included to elucidate more precisely aspects related to functioning in families of children with ASD.

## Conclusion

This study provides important data regarding the daily routines and experiences of families with children with ASD and clarifies the direct impact of symptoms of ASD on them. It provides a foundation for further studies that including larger and more representative samples of families with children diagnosed with ASD. The results may also have implications for the development of more effective programs to support better coping and improved functioning of affected families in developing countries.

## Data Availability

The data that support the findings of this study can be requested from the corresponding author, M. Cordeiro, upon reasonable request based on Data Transfer Agreement.
